# Resolutions of the Benevolent Medical Society

**Published:** 1801-08

**Authors:** 


### *Resolutions of the Benevolent Medical Society.*

RESOLVED,

1ſt. THAT the thanks of this court be given to Dr. Jenner, for his invaluable Treatiſe on the Variolæ Vaccinæ, wherein he has clearly and ſatisfactorily demonſtrated, that the inoculated Cow-pox is a certain preventive of the Small-pox.

That as men of humanity, aſſociated for the purpoſes of benevolence, we ſhould be wanting to the character we aſſume, did we neglect the preſent opportunity of bearing our teſtimony to the value of this providential diſcovery, which, if generally practiſed, we are of opinion, would effectually eradicate the Small-pox, one of the ſevereſt ſcourges of the human race.

That this court, in thus requeſting Dr. Jenner to accept their unanimous thanks for his ineſtimable publication, entertain no doubt but poſterity* will do honour to his memory, and record his name amongſt the real friends of man.

2d. That the reſolution now unanimouſly paſſed, be fairly tranſcribed, and ſent to Dr. Jenner by the chairman, and a copy of it ſent for inſertion in the Medical and Phyſical Journal, and alſo in the Chelmsford and County Chronicles, and that the names of the members preſent be added thereto.

BENEVOLENT MEDICAL SOCIETY for the Counties of Eſſex and Herts. Court of Audit, holden at Dunmow, June 8, 1801.

Preſent,

| | | | |
|---|---|---|---|
| John Andree, M. D. | Surgeons. | Mr. Gretton, | Surgeons. |
| Mr. Burr, | | Mr. Barlow, | |
| Mr. Bradley, | | Mr. Willſher, | |
| Mr. Cribb, | | Mr. Boodle, | |
| Mr. Clarence, | | Mr. Seymour, | |
| Mr. Lawrence, | | Mr. G. A. Gepp, | |
| Mr. Cooper, | | Mr. Harriſon, | |
| Mr. Richard James, | | Mr. Butler, | |
| Mr. John James, | | Mr. Farr, | |
| Mr. James James, | | Mr. J. Clarance, | |

Mr. NEWELL, Chairman.

The foregoing reſolutions, we underſtand, were tranſmitted to Dr. Jenner in a very handſome letter written by Mr. Newell the Chairman.

---

* We not only hope that poſterity will pay this tribute to Dr. Jenner, but that his contemporaries alſo will not be unmindful of his merit. ED.

